# Evaluation of Dwarf Tomato Germplasm for Dual-Purpose Use: Breeding and Rootstock Potential

**DOI:** 10.3390/ijms27219540

**Published:** 2026-10-25

**Authors:** Gabriel Mascarenhas Maciel, Ana Luisa Alves Ribeiro, Lucas Medeiros Pereira, Hugo Arantes Mendonça, Luis Felipe Villani Purquerio, Nádia Nardely Lacerda Durães Parrella, Amilton Ferreira da Silva, Ana Carolina Silva Siquieroli, Orlando Ribeiro de Oliveira, Vinicius Augusto Pereira, Filipe Gomes Barbosa

**Affiliations:** 1 Departamento de Ciências Agrárias, Universidade Federal de São João del-Rei, Sete Lagoas CEP 35701-970, MG, Brazil; analuisa.ribeiro@outlook.com (A.L.A.R.); nadia@ufsj.edu.br (N.N.L.D.P.); amiltonferreira@ufsj.edu.br (A.F.d.S.); filipe.fg.197@gmail.com (F.G.B.); 2 Instituto de Ciências Agrárias, Universidade Federal de Uberlândia, Rodovia BR 050, Km78, s/n, Uberlândia CEP 38410-337, MG, Brazil; lucasmedeiros10394@gmail.com; 3 Instituto de Ciências Agrárias, Universidade Federal de Uberlândia, Rodovia LMG 746, Km01, s/n, Monte Carmelo CEP 38500-000, MG, Brazil; hugo.mendonca1@ufu.br (H.A.M.); orlando.oliveira@ufu.br (O.R.d.O.); vinicius.pereira2@ufu.br (V.A.P.); 4 Instituto Agronômico (IAC), Centro de Horticultura, Campinas CEP 13075-630, SP, Brazil; felipe.purquerio@sp.gov.br; 5 Instituto de Biotecnologia, Universidade Federal de Uberlândia, Rodovia LMG 746, Km01, s/n, Monte Carmelo CEP 38500-000, MG, Brazil; carol@ufu.br

**Keywords:** *Solanum lycopersicum* L., climate change, dwarfing genes, sustainable production

## Abstract

Tomato plant (*Solanum lycopersicum* L.) productivity is threatened by biotic and abiotic stresses, exacerbated by climate change. The use of rootstocks is an effective strategy to enhance tomato resilience. Interest in characterizing dwarf tomato germplasm has increased, though its potential as a rootstock remains unexplored. The objective was to validate the use of dwarf tomato germplasm for: (1) characterizing dwarf tomato genotypes for morphoagronomic traits relevant to hybrid development and (2) evaluating their potential as rootstocks. Two greenhouse experiments were conducted. Experiment I assessed a recurrent parent (normal-architecture tomato), a donor parent (dwarf tomato), and twelve dwarf tomato lines. Morphoagronomic traits of fruits, shoots, and roots were evaluated, allowing the selection of the best dwarf tomato candidate for rootstock use. Experiment II was performed after grafting onto the selected dwarf tomato rootstock (UFU MC TOM1) from Experiment I. Commercial hybrids (Xanthus, Luan, Vivacy, and Bento) were used as grafting material, resulting in four graft combinations: Vivacy/UFU MC TOM1, Xanthus/UFU MC TOM1, Luan/UFU MC TOM1, and Bento/UFU MC TOM1. Both experiments followed a randomized block design in a greenhouse. Data were analyzed using univariate and multivariate approaches. The evaluated dwarf tomato germplasm exhibited relevant morphoagronomic traits, standing out as a promising source for hybrid development. The use of dwarf tomato genotypes as rootstocks proved viable, as grafting significantly increased root volume, overcoming potential limitations associated with the dwarf plant’s root system. These findings highlight the potential of dwarf tomatoes for both breeding programs and grafted seedling production, contributing to crop sustainability and efficiency.

## 1. Introduction

The current climate scenario has directly impacted tomato (*Solanum lycopersicum* L.) productivity worldwide. Available commercial cultivars lack sufficient resilience to diverse biotic and abiotic stresses, highlighting the need for improved genetic breeding strategies. In this context, grafting onto rootstocks has emerged as a viable approach to enhance resistance to biotic and abiotic stresses [1,2].

The use of rootstocks is well-established in various regions worldwide [3]. Their key characteristics include broad-spectrum resistance to soil-borne pathogens, enabling growers to maintain production in pathogen-infested areas [4]. Of particular concern is the expanding geographic range of soil pathogens driven by climate change, which forecasts increased risks of food insecurity in coming decades [5]. This trend is already resulting in significant yield reductions and grower discouragement [3].

The magnitude of research on tomato rootstock utilization was comprehensively assessed by Grieneisen et al. [4], who identified dozens of publications on the subject, reporting over two hundred distinct rootstock genotypes evaluated across more than one hundred global locations. In addition to rootstocks derived from wild tomato accessions [3], alternative approaches such as grafting tomato onto potato rootstocks have also been documented [6], among other innovative combinations. Despite the increasing advances and demonstrated benefits of grafting techniques in tomato cultivation, no published studies have evaluated the use of dwarf tomato germplasm as rootstocks. Several dwarf tomato accessions have been described as having potential for use in breeding programs [7,8]. These accessions represent a valuable breeding strategy due to the well-characterized genetic inheritance of dwarfism [9], which enables exploitation of heterosis in hybrids with additional advantages [10].

The characteristics observed in the UFU MC TOM1 accession suggest the hypothesis that dwarf plants could be used as rootstocks. However, no studies have yet characterized the germplasm of dwarf tomato plants in terms of morphological and agronomic aspects for use as rootstocks in commercial hybrids. The main gap lies in the grafting compatibility between dwarf plants (rootstock) and commercial hybrids (grafting), as well as the response of the roots after grafting, since dwarf plants tend to develop a smaller root system.

Given the above, it is necessary to characterize and select morphological and agronomic aspects of dwarf tomato germplasm to support research aimed at achieving higher productivity in hybrids derived from dwarf male parents. Additionally, due to its remarkable resilience, it is essential to evaluate the use of dwarf tomato as a rootstock. Thus, the objective of this research was to characterize dwarf tomato genotypes and assess their potential as rootstocks.

## 2. Results

**Figure 1:**
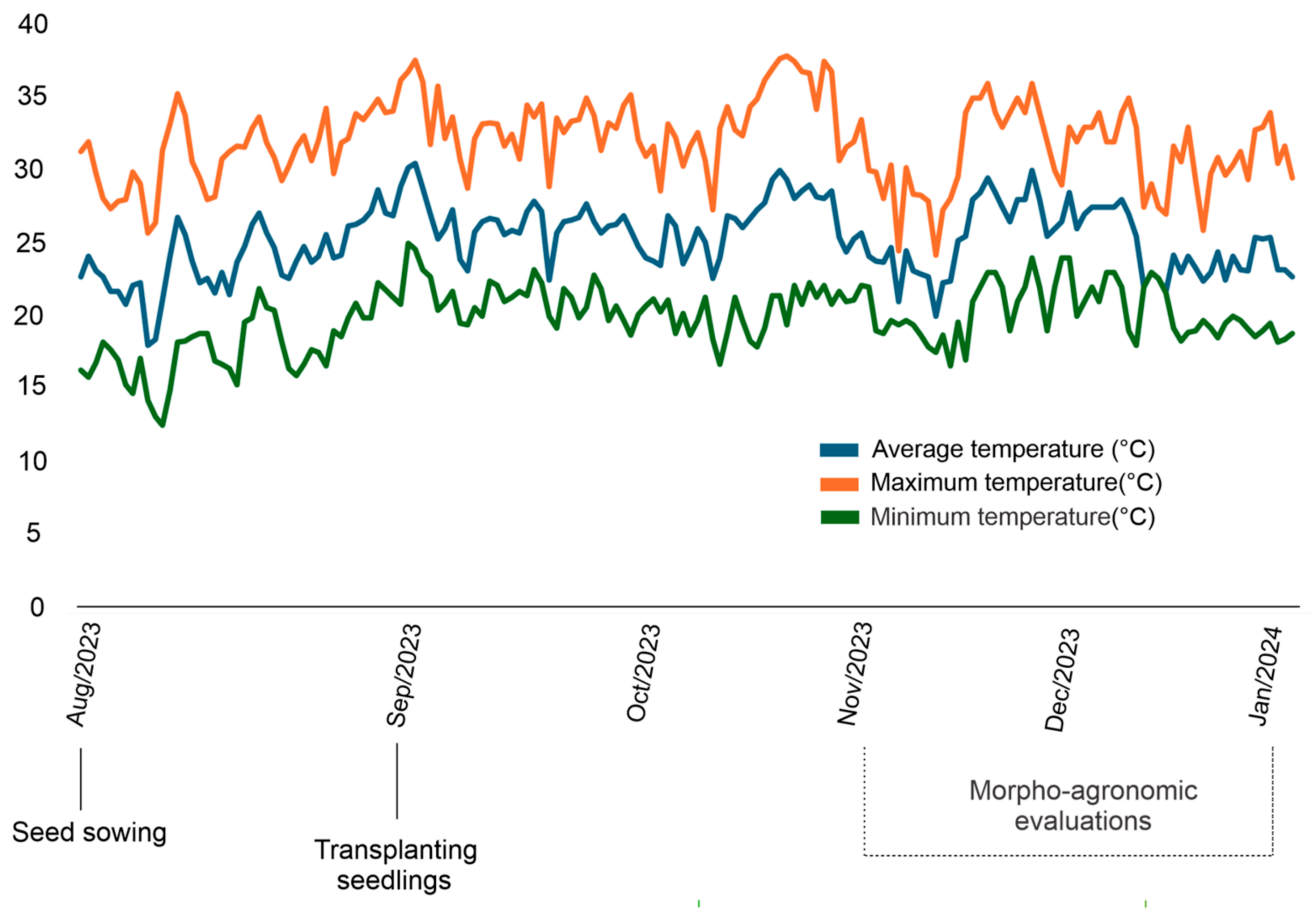
Maximum, Mean, and Minimum Air Temperatures.

The average data for maximum, mean, and minimum temperatures observed inside the agricultural greenhouse are described in Figure 1.

The maximum, minimum, and mean temperatures during the experiment were 37.9 °C, 12.5 °C, and 27 °C, respectively.

### 2.1. Experiment I: Morphological and Agronomic Characterization of Dwarf Tomato Germplasm

**Figure 2:**
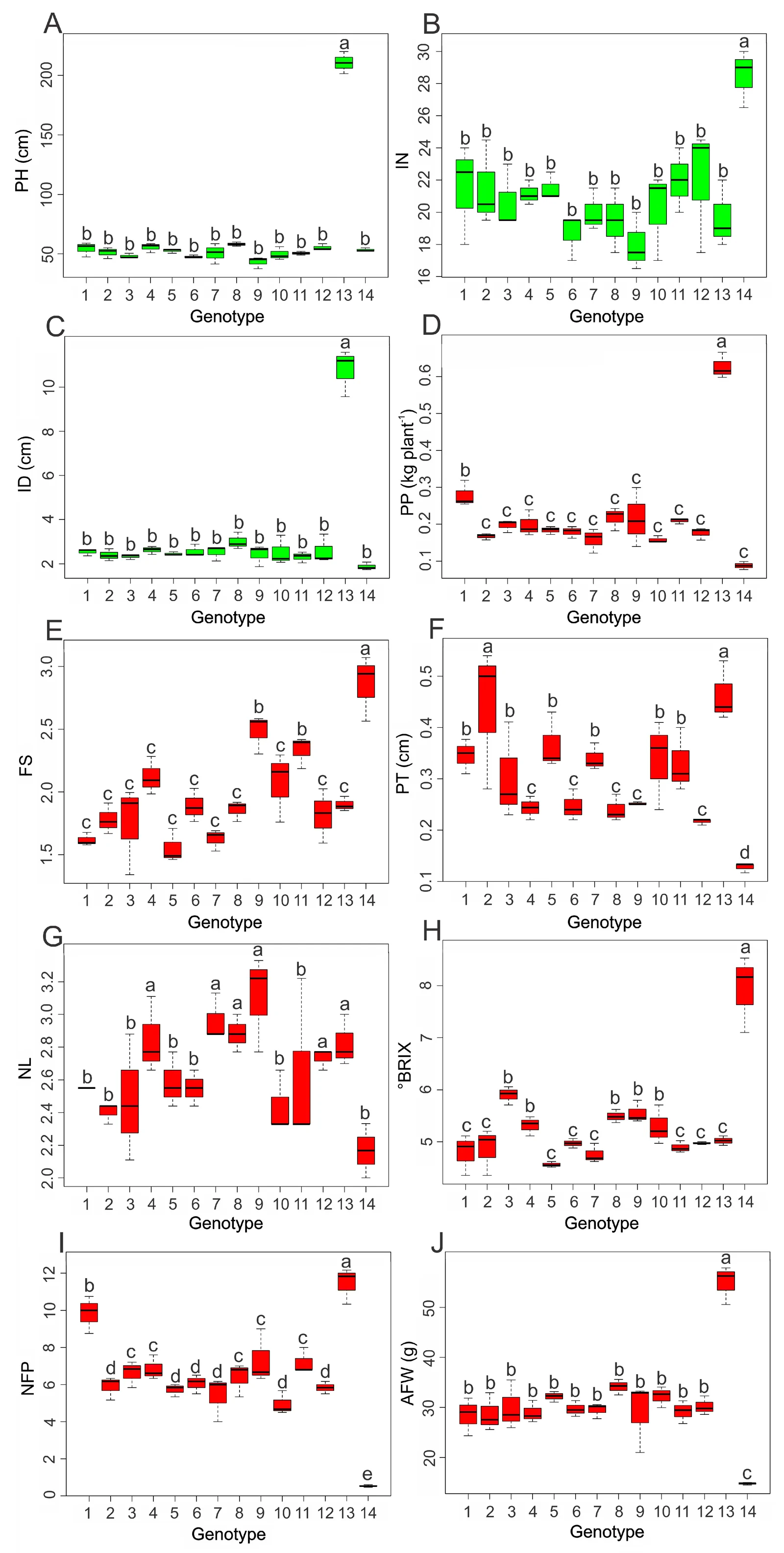
(**A**) Mean values for plant height (PH), (**B**) number of internodes (IN), (**C**) internode length (ID), (**D**) fruit yield per plant (PP), (**E**) fruit shape (FS), (**F**) fruit pulp thickness (PT), (**G**) number of locules (NL), (**H**) soluble solids content in °Brix (BRIX), (**I**) number of fruits per plant (NFP), and (**J**) average fruit weight (AFW) were evaluated. 1: UFU 104511; 2: UFU 104561; 3: UFU 131211; 4: UFU 131221; 5: UFU 174111; 6: UFU 176131; 7: UFU 46111; 8: UFU 104512; 9: UFU 131212; 10: UFU 174112; 11: UFU 176132; 12: UFU 46112; 13: UFU-TOM5, recurrent parent; 14: UFU MC TOM1, donor parent. Means followed by the same letter do not significantly differ according to the Scott–Knott test at a 5% probability level.

Evaluations of morphological and agronomic traits related to plant and fruit morphology showed significant differences (Figure 2).

The morphological characterization of plants (box plot with green coloration, Figure 2A–C) showed that the pre-commercial UFU-TOM5 line (normal architecture) exhibited the greatest plant height compared to all dwarf tomato genotypes. The dwarf tomato genotypes had an average height of 50 cm, while the UFU-TOM5 pre-commercial line reached an average of 200 cm (Figure 2A).

The greater height observed in UFU-TOM5 was associated with a higher IN compared to all dwarf tomato genotypes (Figure 2B). In contrast, most dwarf tomato genotypes exhibited an average internode length of 2.5 cm, which was relatively short. The pre-commercial line (normal architecture) displayed internode spacing four times greater than that of dwarf plants, exceeding 10 cm, which represented a significant difference (Figure 2C).

The pre-commercial UFU-TOM5 line (normal architecture) showed the highest yield per plant, being three times higher than the average yield of dwarf tomato genotypes (box plot with red coloration, Figure 2D–J). Among the dwarf tomato genotypes, UFU 104511 stood out significantly (0.280 kg per plant) as the most productive (Figure 2D).

The fruit shape of most dwarf tomato genotypes did not differ significantly from that of the background parent UFU-TOM5, with values ranging between 1.5 and 2.5, except for UFU MC TOM1, which showed elongated fruits (Figure 2E). Regarding fruit pulp thickness, no significant difference was observed between the dwarf tomato genotype UFU 104561 and the UFU-TOM5 pre-commercial line (4 mm) (Figure 2F). UFU MC TOM1 displayed a thinner pulp, measuring less than 2 mm. The pulp thickness characterization closely resembled the number of locules. UFU MC TOM1, which had the thinnest pulp, also exhibited the lowest number of locules (Figure 2G). On the other hand, UFU MC TOM1 demonstrated strong fruit quality potential, expressing the highest °Brix value, exceeding 8 °Brix (Figure 2H).

The lowest fruit count per plant (Figure 2I) and the lowest average fruit weight (Figure 2J) were observed in UFU MC TOM1 (Figure 2).

**Figure 3:**
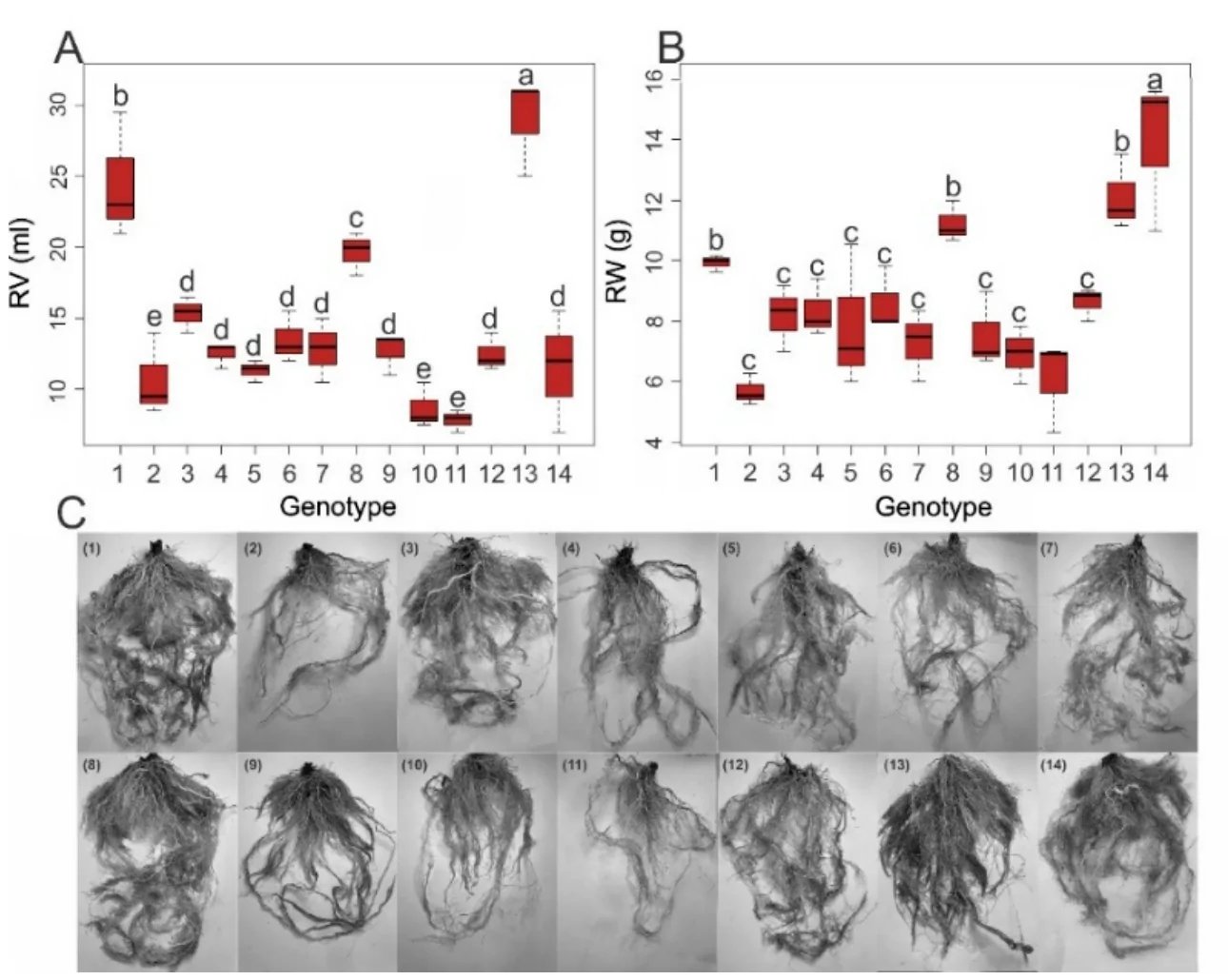
(**A**) = Means referring to root volume in mL (RV) and (**B**) = root fresh mass in grams (RW). Means followed by the same letter do not significantly differ by the Scott–Knott test at 5% probability. (**C**) root systems of the fourteen evaluated genotypes. The description of the treatments is the same as that presented in Figure 2. (**1**): UFU 104511; (**2**): UFU 104561; (**3**): UFU 131211; (**4**): UFU 131221; (**5**): UFU 174111; (**6**): UFU 176131; (**7**): UFU 46111; (**8**): UFU 104512; (**9**): UFU 131212; (**10**): UFU 174112; (**11**): UFU 176132; (**12**): UFU 46112; (**13**): UFU-TOM5, recurrent parent; (**14**): UFU MC TOM1, donor parent.

The characterization of root morphology and variations in magnitude also revealed significant differences in mean root volume (RV, in mL) and root fresh mass (RW, in grams) (Figure 3).

Notably, the UFU 104511, UFU 104512, and UFU MC TOM1 genotypes stood out among the dwarf tomato genotypes considered as rootstock candidates in terms of average root volume, with values of 22.1, 19.8, and 13.9, respectively (Figure 3A), and root fresh mass values of 10.2, 11.3, and 15.1 g, respectively (Figure 3B).

The overall performance results of the dwarf tomato genotypes can be observed in Figure 3C, indicating that UFU MC TOM1 is a promising candidate for compatibility testing and further evaluation to validate its use as a dwarf tomato rootstock.

**Figure 4:**
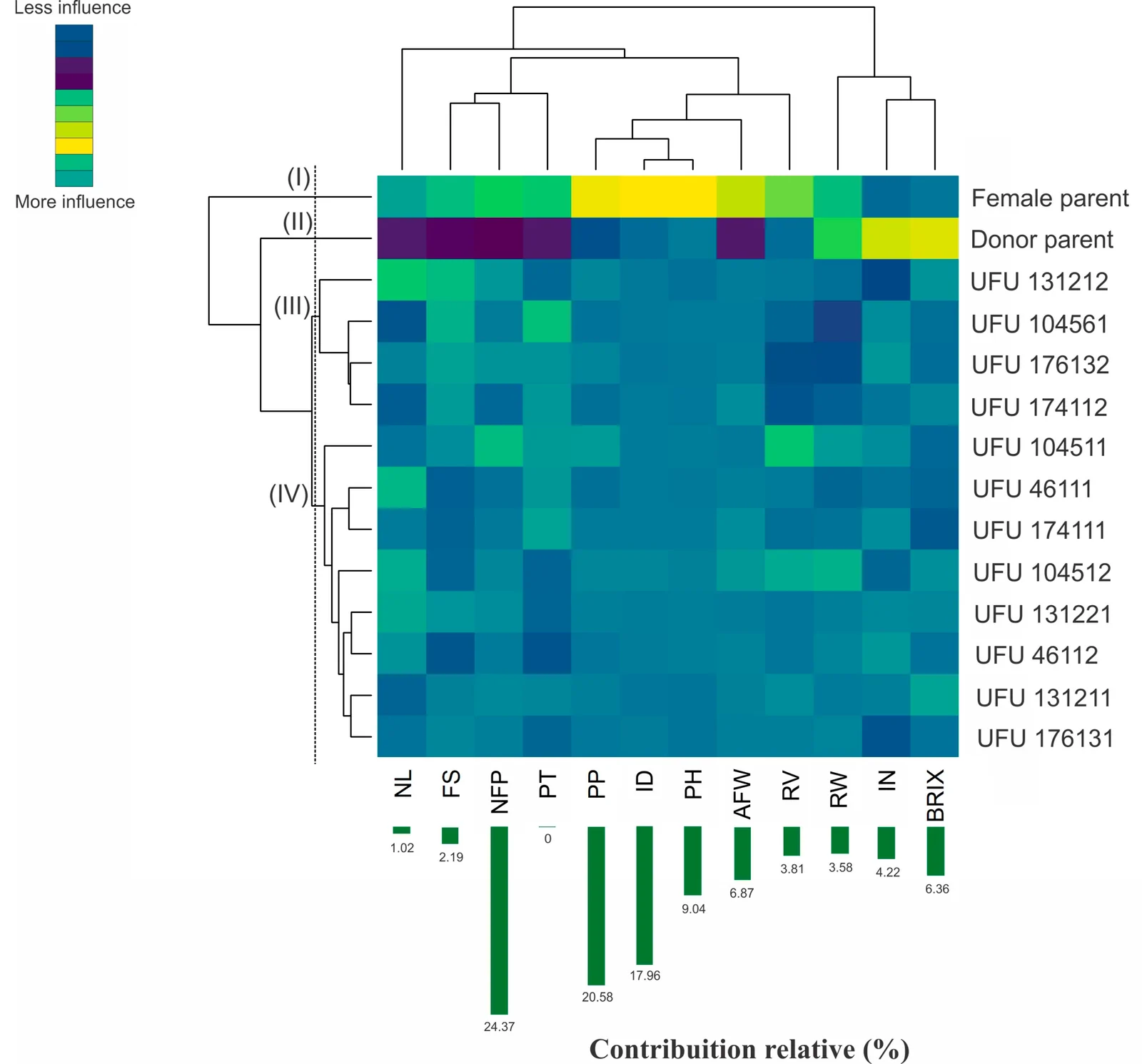
Dendrogram Representing Genetic Dissimilarity Among Tomato Parents and Hybrids, Obtained Using the UPGMA Method. Plant height in cm (PH), number of internodes (IN), internode length in cm (ID), fruit yield per plant in kg per plant (PP), root volume in mL (RV), root fresh mass in grams (RW), fruit shape (FS), pulp thickness in cm (PT), number of locules (NL), soluble solids content in °Brix (BRIX), number of fruits per plant (NFP), and average fruit weight in grams (AFW). Donor parent (UFU MC TOM1); female parent, recurrent parent (UFU-TOM5).

For the genetic dissimilarity analysis, a cutoff point was established in the dendrogram based on abrupt changes in branching patterns, resulting in the formation of four distinct groups (Figure 4).

The cophenetic correlation coefficient (CCC) obtained from the clustering was 99.4%, with 0.78% distortion. Group I revealed the inherent genetic dissimilarity of the pre-commercial normal-architecture line, recurrent parent (UFU-TOM5), and the donor parent (UFU MC TOM1), which was isolated in Group II. The dwarf tomato parents UFU 131212, 104561, 176132, and 174112 were classified as similar in Group III. Group IV comprised the remaining dwarf tomato genotypes.

Notably, the number of fruits per plant contributed the most to the variation (24.4%). Among the dwarf tomato genotypes, UFU MC TOM1 showed the highest root fresh mass (Figure 3B) and, considering the root volume (RV) and root fresh mass (RW) variables (Figure 4), UFU MC TOM1 exhibited characteristics similar to a normal tomato plant (UFU-TOM5), further reinforcing its potential as a candidate for compatibility testing and validation as a dwarf tomato rootstock. These results supported the selection of UFU MC TOM1 as a rootstock for Experiment II.

### 2.2. Experiment II: Use of Dwarf Tomato as Rootstock

Following the grafting of the commercial hybrids Vivacy, Xanthus, Luan, and Bento onto the UFU MC TOM1 rootstock, 100% grafting compatibility was observed. No incidence of bacterial infections was detected in the grafted plants, allowing for an accurate assessment of root development index (g.cm^−3^), relative root volume (cm^3^.cm^−1^), and root/shoot ratio.

**Figure 5:**
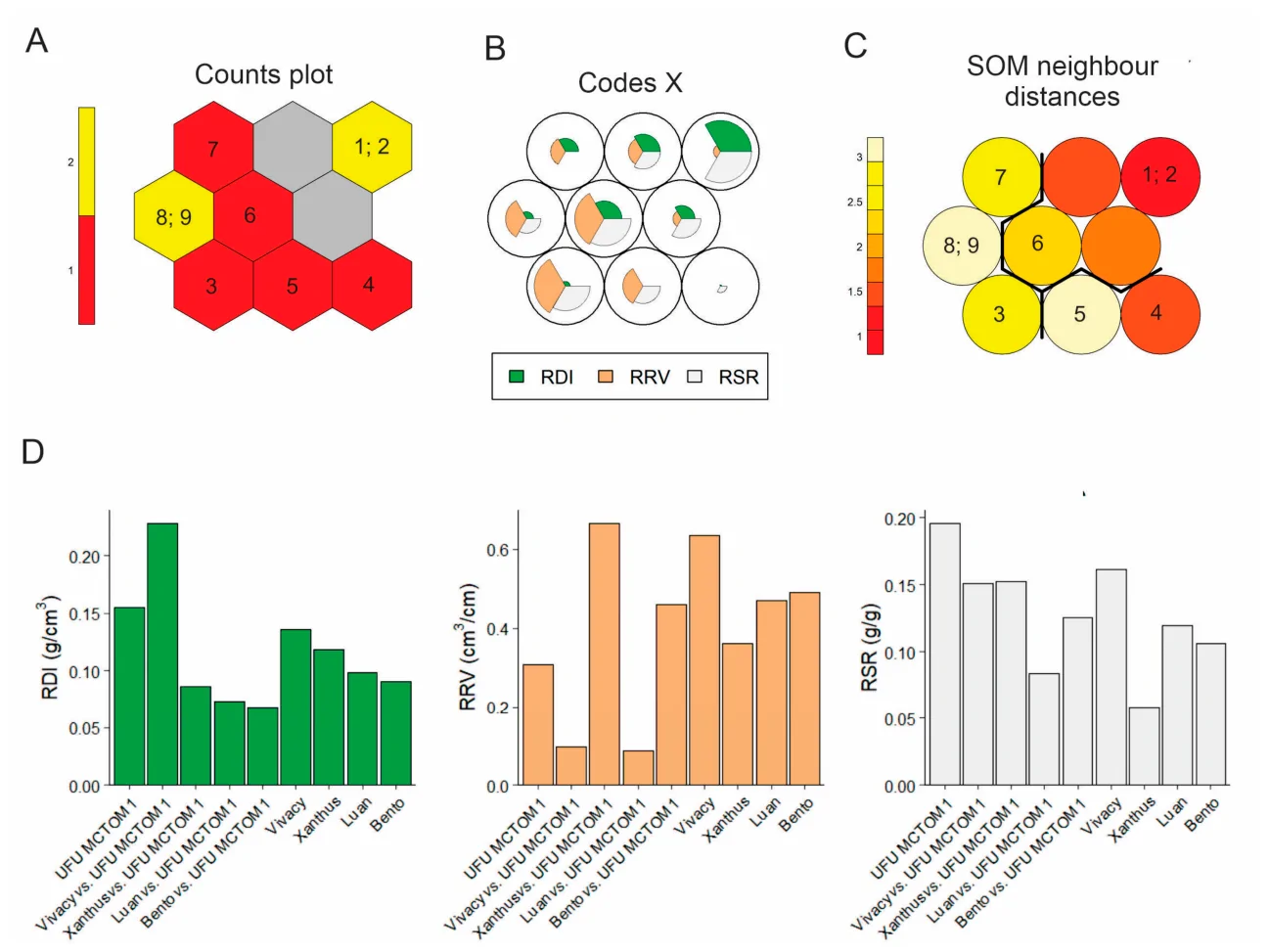
(**A**) Classification of Treatments Based on the Number of Neurons. (**B**) Representation of neurons and the magnitude of variable influence. (**C**) Distances of nearest neighbors and clustering of neurons based on the UPGMA method according to distance. 1: UFU MC TOM1; 2: Vivacy vs. UFU MC TOM1; 3: Xanthus vs. UFU MC TOM1; 4: Luan vs. UFU MC TOM1; 5: Bento vs. UFU MC TOM1; 6: Vivacy; 7: Xanthus; 8: Luan; 9: Bento. (**D**) Root development index (RDI, g/cm^3^), relative root volume (RRV, cm^3^/cm), and root/shoot ratio (RSR, g/g) of the evaluated treatments. Indeed, the UFU MC TOM1 rootstock, as a dwarf tomato, exhibited lower root volume performance. However, after grafting with commercial hybrids, the grafted plants achieved a root volume similar to commercial cultivars (Figure 6).

The genetic dissimilarity analysis comparing the rootstock (UFU MC TOM1), grafting (Vivacy, Xanthus, Luan, and Bento), and grafted combinations (Vivacy vs. UFU MC TOM1; Xanthus vs. UFU MC TOM1; Luan vs. UFU MC TOM1; Bento vs. UFU MC TOM1) was conducted using a self-organizing map based on Kohonen’s method (Figure 5).

**Figure 6:**
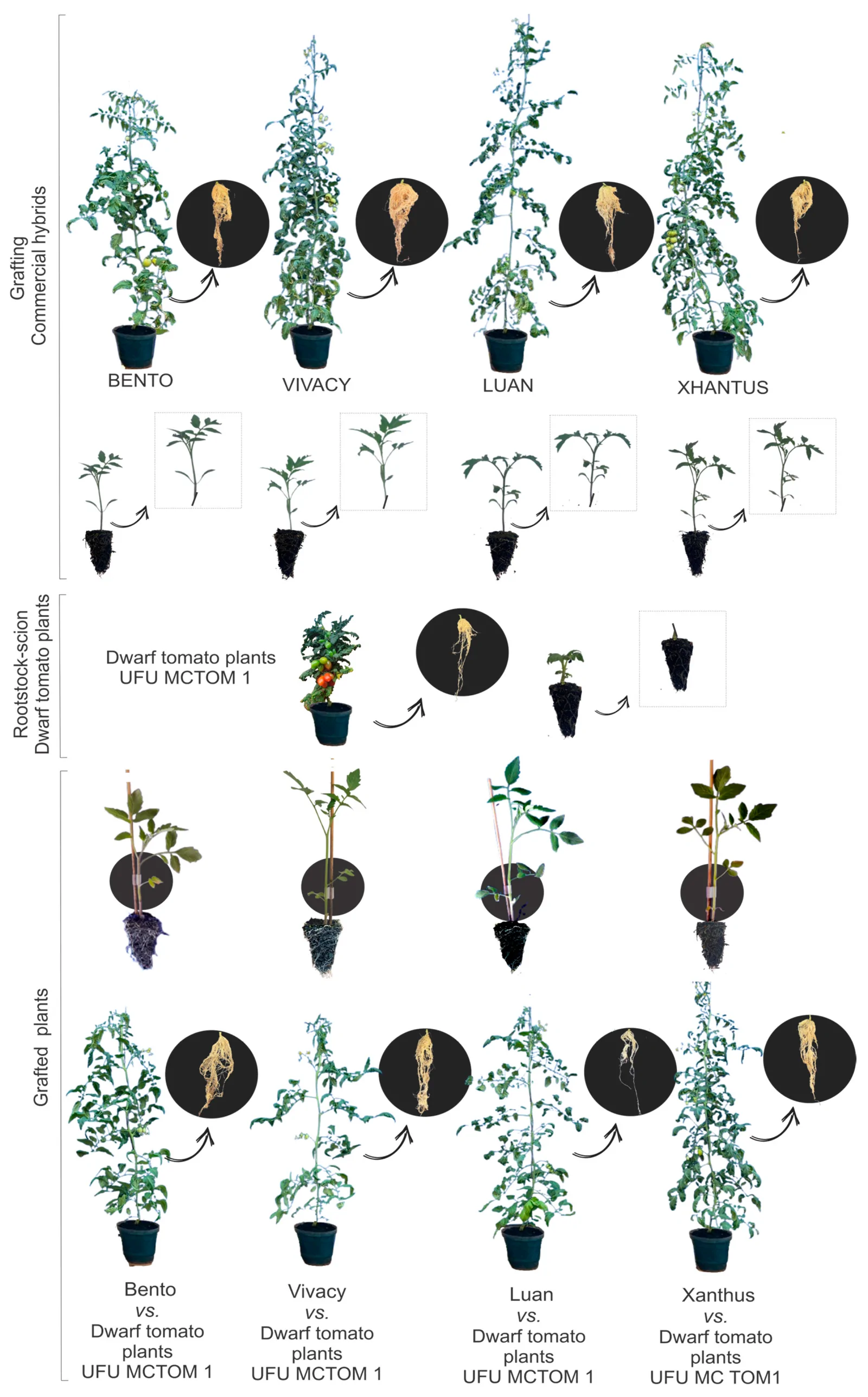
Grafting Scheme with Images of the Commercial Hybrids Used for Grafting, Rootstock UFU MC TOM1, and Roots.

Using the SOM method, an initial set of nine neurons was defined, with two of these neurons not assigned any genotype, resulting in the formation of seven distinct groups (Figure 5A).

The representation of neurons and the magnitude of variable influence highlighted the dwarf tomato genotype UFU MC TOM1, selected as the rootstock, and the hybrid Vivacy (column 3, row 3) (Figure 5B).

Analyzing nearest neighbor distances and clustering of neurons based on the UPGMA method (Figure 5C) made it evident that grafting onto the UFU MC TOM1 dwarf tomato rootstock resulted predominantly in grafted plants with root performance comparable to commercial hybrids and superior to the original dwarf rootstock UFU MC TOM1 (Figure 5C).

## 3. Discussion

Protected cultivation can increase air temperature by 10 to 12 °C compared to external temperatures [11]. The observed temperature values during the experiment ranged from 12.5 °C to 37.9 °C (Figure 1). For tomato cultivation in a protected environment, temperatures between 15 °C and 32 °C are recommended [12]; however, temperatures above 27.5 °C may already reduce the agronomic potential of the crop [13,14]. During the experiment, the mean temperature (27 °C) remained below this threshold (Figure 1).

### 3.1. Experiment I: Morphological and Agronomic Characterization of Dwarf Tomato Germplasm

An analysis of dwarf tomato plant height confirmed that the evaluated germplasm consists of reduced-size plants, presumably carrying the D (DWARF) gene [15]. The D gene is involved in brassinosteroid biosynthesis, and the d locus shortens plants by reducing internode length [16,17]. The inheritance of dwarfism in tomatoes has been studied by Maciel et al. [9] and Finzi et al. [15], confirming a recessive monogenic inheritance pattern for dwarf stature. This mutation is not included in the library of mutations observed by Menda et al. [18] and Saito et al. [19].

The use of dwarf tomatoes has primarily been explored as a model plant for studies. However, their germplasm has also been evaluated for morphological and agronomic traits [8] and for their combining ability in obtaining hybrids with normal plant architecture [10,15]. There are reports that UFU MC TOM1 exhibits high levels of foliar acylsugars [8,20], which could also be present in roots and provide broad-spectrum resistance [21].

The IN and ID have been extensively studied to develop compact tomatoes. Evidence suggests that plant height is regulated by phytohormone interactions [22] or by specific mutations [23]. Additionally, research has shown that the SlGID1a gene can affect internode length [22]. Hybrids derived from dwarf male parents tend to produce more compact and high-yielding plants [10,15]. In the evaluated dwarf tomato germplasm, internodes were shorter, favoring hybrid development, as explored by Finzi et al. [10] and Pereira et al. [15].

Beyond productivity, using dwarf male parents to generate hybrids can enhance fruit quality [15]. In this study, the highest °Brix value was observed in the dwarf donor parent UFU MC TOM1 (Figure 2H), highlighting the potential of dwarf germplasm as a source of fruit quality for future hybrid development.

Multivariate genetic dissimilarity analysis has been successfully applied in breeding programs [10,24] for morphological and agronomic characterization of dwarf tomato germplasm. Indeed, the genotypes were grouped distinctly in the multivariate analysis, maintaining consistency with the univariate analysis (Figure 2, Figure 3, Figure 4 and Figure 5).

Despite advances in dwarfism gene studies for agronomic exploration, a significant gap remains regarding root characteristics in dwarf tomato genotypes. The differences observed in root volume (RV) and root fresh mass (RW) (Scott–Knott test at 5% probability, Figure 3) allowed for the selection of UFU MC TOM1 as a potential rootstock candidate. These findings led to Experiment II, aiming to validate UFU MC TOM1 as a dwarf tomato rootstock, a novel approach in the scientific literature. The selection of UFU MC TOM1 as a rootstock was motivated by its resilience to climatic conditions and its broad-spectrum resistance to pests and diseases [20]. Furthermore, an analysis of the metabolomic profile of UFU MC TOM1 compared to commercial hybrids revealed the presence of key metabolites associated with resistance to various biotic and abiotic stresses [10].

### 3.2. Experiment II: Use of Dwarf Tomato as Rootstock

Compatibility verification is one of the primary criteria for rootstock evaluation [25] (Guerra and Rodrigues 2024). In tomato breeding, significant efforts have been made to validate key rootstocks [3,4,5]. The fact that UFU MC TOM1, a dwarf tomato, achieved high grafting success (%) with four commercial hybrids (Vivacy, Xanthus, Luan, and Bento) opens the door for further evaluations.

Rootstock validation requires compatibility assessment, grafting/rootstock interactions, and agronomic performance evaluation [25]. Given UFU MC TOM1’s dwarf nature, an in-depth analysis was needed to explore potential root system changes after grafting. Remarkably, grafting onto UFU MC TOM1 significantly improved root development index, relative root volume (cm^3^.cm^−1^), and root/shoot ratio in grafted plants compared to the original dwarf rootstock. It is worth noting, however, that these results are limited to early vegetative vigor and initial seedling architecture. A self-organizing map (SOM) analysis identified distinct groupings based on root development index, relative root volume, and root/shoot ratio, comparing rootstock (UFU MC TOM1), grafting (Vivacy, Xanthus, Luan, and Bento), and grafted plants (Figure 5). This multivariate analysis approach has been successfully applied to characterize dwarf tomato germplasm [10].

Grafted plants exhibited superior root performance compared to the dwarf UFU MC TOM1 rootstock (Figure 5D and Figure 6). These results pave the way for future research, allowing further grafting combinations and agronomic evaluations.

## 4. Materials and Methods

### 4.1. Experimental Location and Germplasm

Two experiments were conducted, both taking place at the Experimental Station for Vegetables of the Federal University of Uberlândia (EEH-UFU), located on the Monte Carmelo Campus, MG, Brazil (18° 42′ 43.19″ S, 47° 29′ 55.8″ W, 873 m), between the spring of 2024 and the summer of 2025. The plants were grown in a greenhouse measuring 7 × 21 m with a height of 4 m. The sides were covered with anti-aphid screens, and the cover was made of 200 µm transparent low-density polyethylene (LDPE) film.

### 4.2. Experiment I: Morphological and Agronomic Characterization of Dwarf Tomato Germplasm

Twelve dwarf tomato lines of the Saladette type were evaluated, resulting from hybridization between UFU-TOM5 ♀ (normal architecture) and UFU MC TOM1 ♂ (dwarf). The sowing of the fourteen treatments (the twelve dwarf tomato lines and the two parents, UFU-TOM5 and UFU MC TOM1) was carried out in polystyrene trays filled with commercial coconut fiber-based substrate in the spring of 2024. Forty-five days after sowing (DAS), seedlings were transplanted into 5 L plastic pots. The experiment was conducted using a randomized block design with three replicates, totaling 42 plots. Each plot consisted of six plants arranged in a double row (three plants per row), spaced 0.25 m apart within and between the two rows. Double rows were spaced 1.0 m apart. At 98 DAS, the following traits were evaluated: (i) Plant height (PH): Measured using a millimeter-graduated measuring tape. All plants within the plot were measured from the collar along the main axis to the apex. (ii) Internode number (IN): Counted along the main stem, starting from the first axil in the lower third of the plant up to the last axil in the upper third. (iii) Internode length (ID)*:* Determined by the ratio of total plant height, measured with a tape measure, to the number of internodes. (iv) Fruit production per plant (PP): Calculated as the total fruit weight per plot divided by the number of plants in each plot (kg). (v) *Fruit shape (FS)*: Evaluated based on the ratio of longitudinal to transverse fruit length. (vi) Fruit pulp thickness (PT): Measured using a caliper, with results expressed in cm. (vii) Number of locules (NL): Determined by making transverse cuts in ten fruits, and the average was calculated per plot. (viii) Soluble solids content (°Brix): Measured using an Atago PAL-1 3810 digital refractometer. Juice was extracted from ten fruits, and the average was obtained per plot. (ix) Number of fruits per plant (NFP): The total number of fruits in all plants within the plot was counted, and the average per plant was calculated. (x) Average fruit weight (AFW): Ten representative, mature, intact fruits were collected per plot and weighed using a precision scale. Results were expressed in grams (g). (xi) Root volume: Determined using an indirect method based on Archimedes’ principle [26], measuring water displacement with a 1 L graduated cylinder. (xii) *Root dry mass (g):* Roots were dried in a forced-air circulation oven at 65 °C for 48 h until reaching a constant weight.

### 4.3. Experiment II: Use of Dwarf Tomato as a Rootstock

After completing the aforementioned morphological and agronomic evaluations, the best dwarf tomato candidate for rootstock was selected. The experiment followed a randomized block design with nine treatments: the rootstock (UFU MC TOM1), four commercial hybrids (Xanthus, Luan, Vivacy, and Bento), and four grafting combinations (Vivacy vs. UFU MC TOM1, Xanthus vs. UFU MC TOM1, Luan vs. UFU MC TOM1, and Bento vs. UFU MC TOM1), with three replicates and two plants per plot.

Sowing took place in the summer of 2025 in 200-cell trays filled with coconut fiber-based substrate in an agricultural greenhouse. Grafting was performed 37 days after emergence (DAE), ensuring rootstocks and hybrids had the same stem diameter.

The grafting method involved cutting the rootstock seedlings approximately 5.0 cm above the cotyledons at a 45° angle. The hybrids were trimmed above the first node, retaining two to three leaves (simple English method). Silicone grafting clips (2.5 mm) were used, which were removed on the eighth day post-grafting. During this period, trays were placed in a closed, dark, humid chamber, followed by a five-day acclimatization period with nearly 100% humidity [27].

After acclimatization, seedlings were transplanted and grown in 5 L pots in a greenhouse with a spacing of 0.7 × 0.5 m. Plants were trained with a single stem, and irrigation was performed daily. Cultural practices followed the recommendations of Alvarenga [28].

Fifteen days after grafting, seedlings were evaluated for survival percentage and bacterial wilt symptoms. The grafting success rate (%) was calculated based on the proportion of successfully grafted plants relative to the total grafted plants. At 78 DAS, Root Development Index (g.cm^−3^) was determined as the ratio between root dry mass and root system volume.

(1) $$RDI=\frac{DryMass\;ofthe\;Root\;System\;\left(g\right)}{Root\;System\;Volume\;\left(cm^{3}\right)}$$

Relative root volume (cm^3^.cm^−1^): ratio between the volume of the root system (physical space occupied by the roots) and the plant height (development of the aerial part).

(2) $$RRV=\frac{Root\;System\;Volume\;\left(cm^{3}\right)\;}{Plant\;Height\;\;\left(cm\right)}$$

Root/shoot ratio: relationship between root biomass and shoot biomass.

(3) $$RSR=\frac{Dry\;Mass\;of\;the\;Root\;System\;\left(g\right)}{Dry\;Mass\;ofthe\;Shoot\;System\;\left(g\right)}$$

### 4.4. Statistical Analyses

The data were subjected to assumption verification through variance homogeneity analysis (Lilliefors test), normality analysis (Oneill–Mathew’s test), and additivity analysis (Tukey’s additivity test). After verification, the data were tabulated and analyzed using analysis of variance (ANOVA) via the F-test (*p* < 0.05). Means were compared using the Scott–Knott test (*p* < 0.05). Subsequently, a multivariate genetic dissimilarity analysis was performed among the genotypes using the generalized Mahalanobis distance (“D” _”ii’” ^”2”). Genetic divergence was represented by a dendrogram obtained through the hierarchical Unweighted Pair-Group Method Using Arithmetic Averages (UPGMA) [29]. Additionally, an unsupervised classification of the data was carried out using a self-organizing map (SOM) based on Kohonen’s model [30]. The number of neurons was defined according to the arrangement that provided the best data representation and structure preservation. The selected configuration was 3 × 3, with 4000 iterations for the learning process, a radius of one, and a hexagonal topology. The analyses were performed using the *GENES* software v. 1990.2021.131, integrated with *Matlab* v. R2016a [31] and *R* software v. 4.2.1 [32].

## 5. Conclusions

The evaluated dwarf tomato germplasm presents morphological and agronomic traits that make it a promising source for hybrid development. Additionally, the use of dwarf tomato genotypes as rootstocks appears to be a viable alternative, as grafting significantly increased root volume, eliminating potential limitations related to root system size. These findings highlight the potential of dwarf tomatoes for both breeding programs and grafted seedling production, contributing to sustainable and efficient cultivation.

## Data Availability

Data will be made available on request.
